# Guideline for the Measurement, Analysis and Reporting of Medication Adherence during the Run-in phase of clinical trials (MARMAR)

**DOI:** 10.1186/s13063-026-09717-0

**Published:** 2026-04-16

**Authors:** Non Davies, Bernard Vrijens, Daniel F. B. Wright, Dyfrig A. Hughes

**Affiliations:** 1 Centre for Health Economics and Medicines Evaluation, North Wales Medical School, Bangor University, Bangor, Wales LL57 2PZ UK; 2 AARDEX Group, Liège, Belgium; 3 Department of Public Health, Liège University, Liège, Belgium; 4 Sydney Pharmacy School, Faculty of Medicine and Health, University of Sydney, Sydney, Australia; 5 Department of Clinical Pharmacology & Toxicology, St. Vincent’s Hospital, Sydney, Australia

**Keywords:** Medication adherence, Run-in phase, Run-in period, Lead-in phase, Lead-in period, Single-blind placebo, Enrichment, Delphi study, Delphi method

## Abstract

**Background:**

Trial run-in phases are sometimes used to select participants into clinical trials who are most likely to remain adherent to study medications. The study designs and data analysis methods employed lack clear regulatory guidance while reporting is not consistent or transparent. This study aims to develop a consensus guideline for the Measurement, Analysis, and Reporting of Medication Adherence during the Run-in phase (MARMAR) of clinical trials.

**Methods:**

Initial “items” (specific statements relating to the measurement, analysis, and reporting of medication adherence during trial run-in phases) were generated following a systematic review of trial run-in phases. Items were split into 2 domains (methods and reporting), refined through a pilot exercise and subsequently evaluated using a two-round modified Delphi survey of international experts in medication adherence and clinical trials. Participants were recruited via e-mailing lists. In survey round one, respondents rated the importance of items across a scale labelled strongly agree to strongly disagree and provided free-text comments. In survey round two, respondents categorised items as essential or desirable and suggested alternative wording. A final consensus meeting of the research team was held to finalise the guideline. Guideline development was endorsed by the International Society for Medication Adherence (ESPACOMP) and registered with EQUATOR.

**Results:**

In round one, 49 respondents rated 29 items, with 64–100% (mean 86.5%) of experts scoring an item 4 or 5. Consensus was reached for 28 items, and 226 free-text comments led to merging overlapping items. In round two, 27 respondents rated 20 revised items, with 33–100% (mean 78.7%) of experts judging them essential and providing 66 free-text comments. The final MARMAR guideline comprises 20 items: 9 methodological and 11 reporting considerations for medication adherence during trial run-in phases.

**Conclusion:**

The MARMAR guideline provides the first structured, consensus-based guideline specifically focused on medication adherence during trial run-in phases. By supporting more transparent and rigorous measurement, analysis and reporting, it should serve to improve the quality of future trial run-in phases.

**Supplementary Information:**

The online version contains supplementary material available at 10.1186/s13063-026-09717-0.

## Introduction

Approximately 5% of randomized controlled trials employ a run-in phase [1], a type of enrichment strategy used to screen for placebo responders, washout previous therapy, familiarise participants with study procedures, and assess treatment adherence [2–5]. The goal is to identify participants prior to randomisation who will have the lowest probability of creating unwanted noise that might obscure signals in the data. Run-in trials are particularly important to identify and exclude participants who are unlikely to be adherent to medications prior to the initiation of the main trial. Once the clinical trial begins, medication nonadherence can undermine the assessment of efficacy and harm, whilst impacting the overall costs involved [6]. The inclusion of a trial run-in phase therefore has practical value since poor adherence behaviour in the run-in trial will often predict future poor medication adherence [7]. However, while trial run-ins are used mainly to increase the internal validity of trials, this is at the expense of external validity, as the results may be less generalisable to patients encountered in routine clinical practice [8]. Judicious use of run-ins is necessary especially in the context of whether trials are aimed at demonstrating the efficacy or effectiveness of medicines [4, 7].

Both the United States Food and Drug Administration (FDA)'s guidance for Enrichment Strategies for Clinical Trials [3] and the World Health Organization’s guidance for Best Practices for Clinical Trials [9] make reference to run-ins as an approach to increase the likelihood of adherence to allocated trial intervention. Despite this, there is no published guidance on the optimal study designs or analysis methods to maximise the chance of detecting nonadherent participants in run-in trials from regulatory authorities including the FDA, European Medicines Agency (EMA), and Medicines and Healthcare Products Regulatory Agency (MHRA), and there is no reference to run-ins in the Consolidated Standards of Reporting Trials (CONSORT) statement [10] or its extensions.

The recent update of the Good Clinical Practice (GCP) guideline ICH-E6(R3) specifies that a trial protocol should include “strategies to monitor the participant’s adherence to treatment” (Section B.7.3) [11]. Although further details are not provided, this highlights the growing recognition of adherence as a critical factor in ensuring reliable and valid clinical trial outcomes [6].

Previous reviews have characterised the frequency and reporting of run-ins [1, 12], and have examined the assesment and reporting of medication adherence in later-phased trials [13–15]. A recent systematic review noted the poor characterisation and reporting of medication adherence during the run-in phase of phase 2 and 3 drug trials, with the methods used often at high risk of bias [16]. The findings of this review coupled with the lack of regulatory guidance highlight the need for a guideline on best practice for the measurement, analysis and reporting of medication adherence during the run-in phase of trials. This study aims to develop a guideline on the optimal methods used to measure and report medication adherence during the run-in phase of trials.

## Methods

### Overview

A pilot study was conducted to obtain feedback on both the draft guideline items and survey design. The term “items” refers to specific statements relating to the measurement, analysis, and reporting of medication adherence during trial run-in phases. These were split into 2 domains—items focused on methods, and those focused on reporting. Items were then presented to invitees of the first round of the Delphi study. Based on feedback from this round, items were refined, with some combined or removed, before progressing to the second Delphi round, where further revisions were made in response to participant feedback. A final consensus meeting of the research team was then held to finalise the MARMAR guideline.

### Registration and protocol

The protocol for this study is registered with the Open Science Framework (https://osf.io/hctxf), and endorsed by the International Society for Medication Adherence (ESPACOMP) (https://www.espacomp.eu/request-for-espacomp-endorsement/). The guideline was also registered as a guideline under development with the Enhancing the Quality and Transparency Of health Research (EQUATOR) network (https://www.equator-network.org/library/reporting-guidelines-under-development/reporting-guidelines-under-development-for-clinical-trials/#MARMAR).

### Pilot study

A convenience sample of 12 work colleagues and close associates/collaborators who were familiar with methodological health research was selected by the research team and recruited via email. Participants were asked to assess validity by providing feedback on both the draft guideline items and the survey design.

### Development of the initial items

The initial items for the Delphi study were informed by a systematic review on adherence in trial run-in phases [16]. The ESPACOMP Medication Adherence Reporting Guideline (EMERGE) guideline was also used to inform the reporting items, despite recognising that the interpretation of EMERGE in the context of run-ins is limited.

### Delphi study

The Delphi method is the preferred approach for developing reporting guidelines [17]. This involves gaining expert consensus through iterative survey rounds, while maintaining participant anonymity [18, 19]. The Delphi method has been applied previously in adherence research, including in the development of EMERGE, which provides general reporting guidance for medication adherence in clinical research [18], the development of a core outcome set for interventional adherence studies of medication users for long-term health conditions [20], and the development of a core domain set for evaluating medication adherence interventions among individuals with rheumatic disease [21].

The Delphi process was conducted following the RAND methodological guidance [22] and reported in accordance with the Delphi studies in social and health sciences — Recommendations for an interdisciplinary standardized reporting (DELPHISTAR) guidance [23], to enhance methodological rigour, transparency and reproducibility.

#### Participants

The Delphi study included subject-area experts and Patient and public involvement (PPI) contributors who had self-declared as having ≥ 5 years' experience in any of the following: medication adherence research, trials methodological research or regulatory drug trials, and who were able to read and write in English.

#### Patient and public involvement

PPI contributors were identified for participation via the emailing lists described, as some were known to include patient and public members.

#### Recruitment

Participants were recruited via the emailing mailing lists of the Trials Methodology Research Partnership (TMRP), ESPACOMP, the NIHR Statistics Group, the United Kingdom Clinical Trial Collaboration Clinical Trial Units (UK CRC CTUs), Statisticians in the Pharmaceutical Industry (PSI), and the UK-based worldwide e-mail discussion list for the statistical community, ALLSTAT. This was facilitated by administrators within each organisation following formal requests for permission. Individuals identified from relevant publications were also contacted via email.

All surveys were administered online via an embedded URL link within the body of the email, using the Jisc Online Surveys platform. The e-mail invitation included:

- A brief description of the study and the aim of the survey.
- A participant information sheet (as an URL) with more details of the survey and data protection policy.
- A URL link to the survey – the first page of which sought consent.
- Details of the ethical approval.
- A contact name, details and e-mail address.
- Acknowledgement of the study funder

#### Informed consent

Progress beyond the landing page of the survey confirmed participant consent.

#### Ethical approval

Ethical approval for the Delphi study was granted by Bangor University’s School of Medical and Health Sciences Academic Ethics Committee (Reference number: 0469).

#### Round 1

During round 1, participants were asked about their employer type and profession, country, and years of experience. Participants were also asked to provide their e-mail address in order to be contacted for round 2. During round 1, participants were asked to rate the importance of each item using a 5-point Likert scale, labelled ‘strongly disagree’ to ‘strongly agree’. Participants were also able to suggest modifications to items or propose new items in free text during round 1. One e-mail reminder was issued 2 weeks after the first e-mail invitation.

#### Round 2

Round 1 participants who provided their e-mail addresses and consent to be contacted were invited to a second round which was assumed to be necessary to achieve consensus. As it transpired, consensus was achieved after round 1. We therefore repurposed round 2 to focus on refining item wording and prioritising items for qualitative validation. As such, participants were asked to rate whether they thought each item was essential or desirable, and whether they were happy with the wording (with an opportunity to provide further comments). The final guideline was planned to highlight a small number of items deemed to be ‘essential’, as in the EMERGE guideline [24]. One e-mail reminder was issued 2 weeks after the first email invitation.

#### Defining consensus

Our definition of consensus aligned broadly with that previously used to develop the EMERGE guideline [18]. Consensus on the relevance of an item was defined as ≥ 70% of the experts in the sample giving an item a score of ≥ 4 on a 5-point Likert scale during any survey round. Items where consensus was met were kept on the final item list. Consensus on the irrelevance of an item was defined as ≥ 70% of the participants giving this item a score of ≤ 2 during any survey round. These items were removed from the final item list.

#### Managing new items/merging existing items

After each survey round, the research team met to discuss any revisions to items, the inclusion of any new items, or the merging of existing items.

#### Stopping criteria

The stopping criterion for this study was consensus achieved after a pre-specified number of rounds, as recommended by the RAND network methodological guidelines for conducting Delphi studies [22]. This was anticipated to be 2 rounds, but was stated not to exceed 3.

#### Data management

All data was collected and managed within Jisc, but also transferred to Bangor University and analysed in Microsoft Excel.

#### Data analysis

Data were analysed descriptively and included a summary of the Likert scale responses and response rates for each round. No statistical inference testing was performed.

## Results

### Pilot study

Nine individuals agreed to participate in the Pilot study. Based on the feedback received, minor revisions were made to the initial guideline items. No changes were required to the survey design, and no new items were suggested or removed.

### Delphi study participants

Forty-nine participants from 17 countries were recruited to the study (Table 1). The majority were involved in clinical trial methodological research (37 participants) and/or medication adherence (33), while 9 were involved in regulatory drug trials. The modal category for experience was 10–20 years (17 participants), and for employment was academic or research institutions (39 participants). Participants represented a multidisciplinary panel, including statisticians and data scientists (n = 12), clinical experts (n = 14), psychological/behavioural experts (n = 3), academic researchers (n = 16), patient and public contributors (n = 3), and industry representatives (n = 1).

**Table 1:** Participant characteristics

Characteristic	Category	*n* (%)
**Continent**	Europe	35 (71.4)
	North America	11 (22.4)
	Oceania	2 (4.1)
	Africa	1 (2.0)
**Expertise**	Trials methodology	37 (75.5)
	Medication adherence	33 (67.3)
	Drug regulatory trials	9 (18.4)
**Profession**	Statistician/biostatistician/data scientist	12 (24.5)
	Clinical expert	14 (28.6)
	Psychological/behavioural expert	3 (6.1)
	Academic/research (non-clinical)	16 (32.7)
	Patient and public contributor	3 (6.1)
	Industry/private sector	1 (2.0)
**Experience (years)**	5–10	16 (32.7)
	10–20	17 (34.7)
	20–30	10 (20.4)
	> 30	6 (12.2)
**Employer type**	Academic or research institution	39 (79.6)
	Non-profit or patient advocacy organization	3 (6.1)
	Healthcare or clinical setting	2 (4.1)
	Both academic and healthcare settings	2 (4.1)
	Pharmaceutical or biotech industry	2 (4.1)

### Round 1

**Fig. 1:**
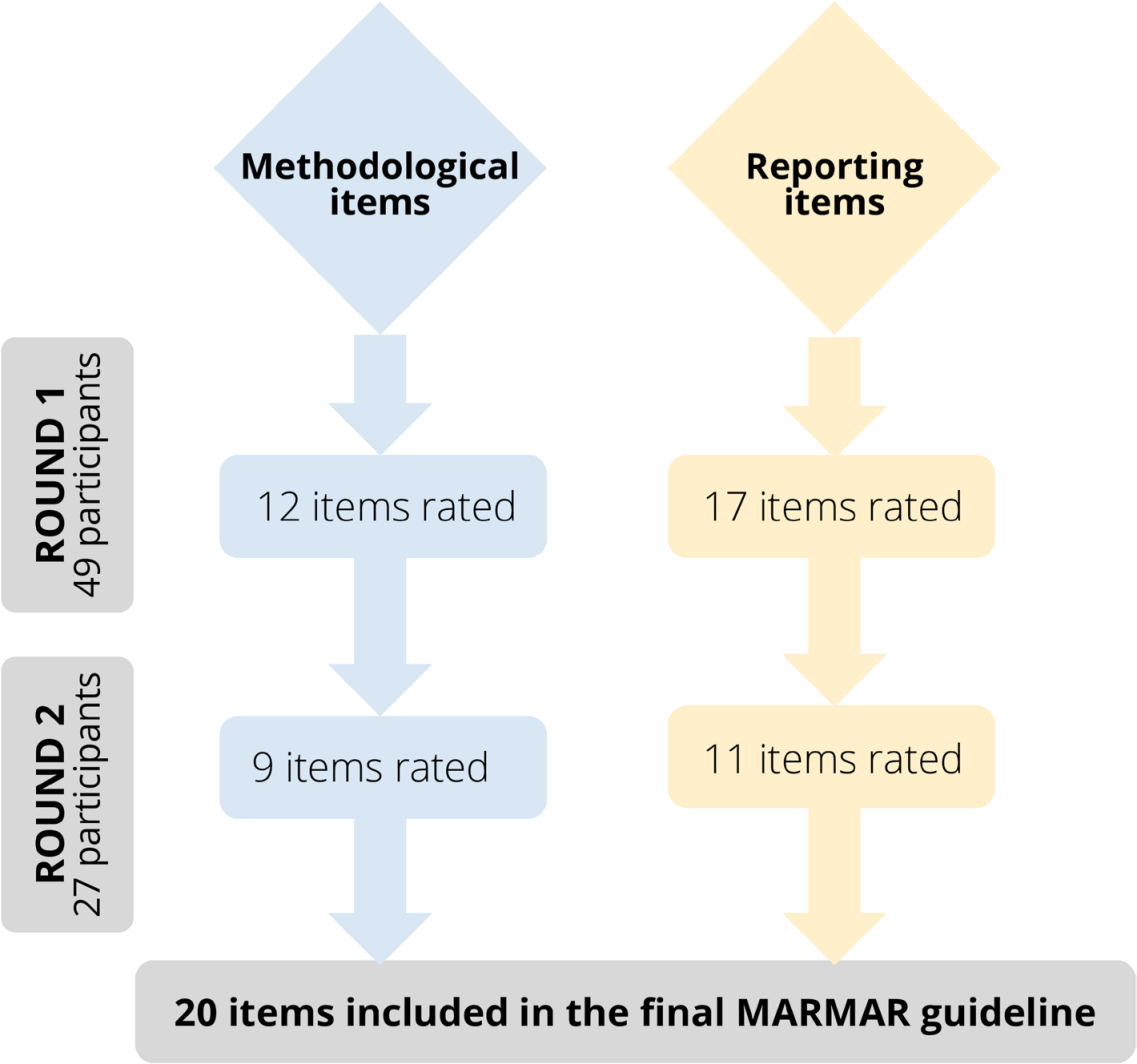
Delphi study process

The Delphi study process is illustrated in Fig. 1. Round one from 27/05/2025 until 27/06/2025, with 49 participants rating 29 items (12 method items and 17 reporting items). The percentage of experts scoring an item ≥ 4 ranged from 64 to 100% (mean 86.5%). Consensus was achieved for 28 out of the 29 items, whilst dissensus remained for 1 item. A total of 226 free-text comments were received.

### Round 2

The second online rating round ran from 07/07/2025 until 07/08/2025, with 27 out of the 41 participants invited from the first survey round participating in the second survey round (66% response rate). Participants were asked to rate 20 items (9 method items and 11 reporting items). The percentage of experts scoring an item as essential ranged from 33 to 100% (mean 78.7%), and 66 free-text comments suggesting alternative wording were received.

### Final consensus meeting

After all survey rounds were complete, the research team met to finalize the guideline items. The discussion focused on refining both the wording and the ordering of items. Table 2 provides examples of how guideline items evolved and the key feedback that prompted each revision.

**Table 2:** Evolution of Delphi item wording

	**Delphi round 1**	**Delphi round 2**	**Final item**
Example 1 (Methods domain: Adherence-based eligibility)			
Item wording	“If the run-in is used to select adherent participants for the main trial, justify the clinical validity of the eligibility criterion by which participants are defined as adherent”	“If the run-in is used to select adherent participants for the main trial, explain the criterion used to define participants as adherent”	“If the run-in phase is used to identify participants who are adherent, clearly specify the adherence criteria that determine eligibility for inclusion in the main trial”
Key feedback	"Clinical validity" is unclear and needs clarification	"Explain" is ambiguous; consider "justify" or "describe"	—
Example 2 (Methods domain: Data management)			
Item wording*	“Individual patient medication adherence data from the run-in phase should be monitored centrally (meaning the remote evaluation of data quality to help ensure its integrity)”	“Individual patient medication adherence data from the run-in phase should be monitored centrally (meaning the remote evaluation of data quality to help ensure its integrity)”	“Individual-level adherence data collected during the run-in phase should be treated consistently with other trial data, in accordance with the most recent ICH Good Clinical Practice Guideline (E6 R3 or future iterations)”
Key feedback	"Centrally" unclear, might not always be feasible	Difficult to understand this item, avoid using "should"	—

^*^ Item wording retained in round 2 for further feedback

### The final MARMAR guideline

The MARMAR guideline items are split into two domains (methods and reporting), and presented with accompanying guidance in Tables 3 and 4, respectively. A template of the guideline in editable Microsoft Word (.docx) format is available in the Supplementary Appendix. The methods domain addresses 9 methodological considerations, and the reporting domain addresses 11 reporting considerations. Several items have overlapping themes, wording and structure given that many of the methodological considerations also need to be reported. As more than half of the items (10 out of 19) were rated as essential by ≥ 80% of participants, highlighting a small number of items deemed to be ‘essential’ was impractical.

**Table 3:** Guidance for using the MARMAR methods domain

Theme	Item	Guidance
1. **Adherence-Based Eligibility**	If the run-in phase is used to identify participants who are adherent, clearly specify the adherence criteria that determine eligibility for inclusion in the main trial	Run-ins are sometimes used to identify adherent participants for inclusion in the main trial. The criteria for defining participants of the run-in as being adherent should be outlined clearly, and based on a pre-specified, objective metric. If an adherence threshold is used, it should be supported by evidence showing that the chosen threshold predicts main trial adherence. Vague statements such as “adherent participants will proceed to the main trial” should be avoided
2. **Objective Progression Criteria**	Decisions to progress participants from the run-in phase to the main trial based on medication adherence should be guided by a pre-specified metric, rather than left solely to investigator discretion	Investigators should not use their own discretion when selecting which participants on the basis of adherence proceed with the main trial, as this introduces a potential source of bias
3. **Appropriate Measurement Methods**	The method(s) used to measure adherence during the run-in should be appropriate to the phase of adherence being studied, the dosing regimen, and the mode of administration	Different methods may be appropriate for measuring whether or not participants initiate trial investigational medicinal products compared to the extent to which participants’ actual dosing correspond to the prescribed dosing regimen (see the ABC taxonomy of adherence for further details https://doi.org/10.1111/j.1365-2125.2012.2012.04167.x). The choice of measurement should also be tailored to the route of administration (e.g. oral, parenteral, inhaled, transdermal, topical). For example, electronic monitoring is useful for measuring the extent to which participants’ actual dosing corresponds to the prescribed dosing regimen as it can register the date and time a medication bottle is opened. However, electronic methods may be impractical for some delivery routes, such as transdermal administration, where alternative approaches, such as patch counts, may be more appropriate*.* Medication adherence measurement methods should also account for timing in relation to dietary requirements, as deviations can influence both adherence and treatment outcomes. For example, when a medicine must be taken on an empty stomach, self-reported measures of adherence such as questionnaires may better capture this
4. **Risk of Bias and Imprecision**	When selecting an adherence measurement method(s) for the run-in phase, assess the potential for bias and imprecision in relation to factors such as acceptability, practicability, feasibility, and cost. Consider risks such as low sensitivity/specificity, recall bias, and pill dumping	For example, the measurement of drug or metabolite concentrations in blood or urine are accurate and reliable methods, but are expensive and invasive, whilst often failing to account for individual differences, including in drug metabolism. Performing pill counts is easy and inexpensive but may be unreliable due to practices such as “pill dumping”, in which patients discard their medication before a clinic visit in order to appear more adherent. Electronic monitoring is precise but provides an indirect estimate of adherence and may not be inclusive of all forms of medication delivery systems
5. **Impact of adherence monitoring on participant behaviour**	Consider whether the chosen adherence measurement method(s) might itself influence participant behaviour (i.e. the Hawthorne effect)*,* and account for this in the trial design	Some adherence measurement methods may make participants aware that their adherence is being monitored, to the extent that it may impact their medication-taking behaviour. Whilst it may not always be possible to avoid this, the impact should be minimised where possible, such as by not providing participants with feedback of their adherence records when using electronic monitoring methods
6. **Observation Period**	Justify the duration of the observation period over which adherence is measured during the run-in	Run-ins are typically of short duration, typically lasting about 2 weeks. It is important to consider the validity of the duration of the run-in period in relation to factors such as its ability to predict adherence in the main trial and dosing regimen. For example, measuring adherence to a once weekly dosing regimen over a 2 week run-in would only capture adherence to two doses, which is unlikely to adequately predict adherence over a 12-month trial
7. **Adherence Summary Metrics**	Justify the individual-level metric(s) used to summarise adherence during the run-in	As part of this justification, trialists should assess the potential limitations of the metric(s) in relation to the phase of adherence being studied, the method of measurement, and considering that run-ins are typically of short duration. For example, while proportion of days covered (PDC) can be easily calculated from raw pill counts and it can overestimate adherence if medication is collected but not actually taken, and is sensitive to missed doses, which may be particularly impactful considering the usually short durations of run-ins
8. **Data Management**	Individual-level adherence data collected during the run-in phase should be treated consistently with other trial data, in accordance with the most recent ICH Good Clinical Practice guideline (E6 R3 or future iterations)	The ICH Good Clinical Practice update (E6R3) places a strong emphasis on the quality and integrity of trial data. Medication adherence data collected during the run-in phase should therefore be given the same consideration as any other trial outcome data
9. **Statistical Analysis**	Justify the statistical methods used to analyse adherence data from the run-in phase	The statistical methods chosen to analyse run-in adherence data should be appropriate for the type, distribution, and completeness of the adherence data collected during the run-in

**Table 4:** Guidance for using the MARMAR reporting domain

Theme	Item	Elaboration
1. **Purpose of Adherence Measurement**	Clearly report the purpose of measuring adherence during the run-in phase	Run-in phases are used for different purposes, such as to select adherent participants for the main trial, assess tolerability to treatment, establish baseline incidence or values, washout or eliminate the effects of previous therapy, or familiarise participants with study procedures and techniques. This should be clearly reported *e.g. “This study included a 2-week run-in phase to select adherent participants for the main trial.”*
2. **Eligibility Criterion for Adherence-Based Inclusion**	If the run-in is used to select adherent participants for the main trial, report the specific adherence criterion used to define eligibility	State clearly and unambiguously *e.g. “Participants who are* < *90% adherent to their antiretroviral therapy during the run-in phase will proceed with the main trial. This threshold is supported by prior clinical evidence indicating that adherence of* < *90% is generally required to achieve adequate viral-load suppression.”*
3. **Implications of Adherence-Based Selection**	If adherence during the run-in is used to determine inclusion in the main trial, report the potential implications of this selection	Restricting main trial inclusion to participants who are adherent during the run-in phase can reduce the risk of biased estimates of efficacy (high internal validity), but also compromise the external validity of the trial. This approach results in a selected population that may not reflect typical patients in routine clinical practice, thereby limiting the generalizability of the results. In addition, the interaction between adherence and tolerability should be taken into account. For example, if a participant experiences tolerability issues, these should be recorded as tolerability criteria. However, as such issues may influence adherence, they should be considered when interpreting adherence data e.g. *“Because only participants who met the adherence eligibility criterion during the run-in proceeded with the main trial, the main trial population consisted of highly adherent participants who may not reflect typical patients in routine clinical practice, thereby reducing the external validity of the results*
4. **Adherence Measurement Methods**	Report the method(s) used to measure adherence during the run-in	*e.g. “Adherence will be assessed by measuring the mass of the returned inhaler canister.”*
5. **Limitations and Biases of Measurement Methods**	Report potential limitations of the adherence measurement method(s), including risks of bias and imprecision*,* as well as whether the method may itself influence participants’ adherence behaviour	*e.g. Whilst pill counts are a simple and inexpensive method for assessing medication adherence, we recognize that it is not inclusive to all forms of medication, cannot explain the reasons behind observed behaviours, and may overestimate adherence – for example due to practices such as “pill dumping,” where participants discard remaining medication before a pill count is performed in order to appear adherent*
6. **Observation Period**	Report the duration of the observation period over which adherence was measured during the run-in	The duration of the observation period for measuring adherence during the run-in should be reported to allow the adequacy of the observation period for evaluation adherence behaviour and its relevance to the main trial to be evaluated *e.g. “This trial will include a 2-week run-in phase, during which adherence will be measured.*“
7. **Adherence Summary Metrics**	Report the metric(s) used to summarise adherence at the participant level during the run-in, and assess its limitations	*e.g. “Proportion of days with correct dose (PDCD) will be used to summarise adherence outcomes at an individual level during the run-in phase.”*
8. **Statistical Analysis**	Report the results of the statistical analysis of adherence data collected during the run-in	*e.g. “Based on electronic monitoring, mean (95% CI) PDCD during the run-in was high – 96.7% (93.7%, 99.7%).”*
9. **Participant Flow**	Report the number of participants enrolled into the run-in, excluded following the run-in, and those who proceeded to the main trial. Include this information in the study’s CONSORT diagram	*e.g. “A total of 371 participants were enrolled into the run-in, 118 participants were excluded following participation in the run-in and 253 participants proceeded to the main trial.”*
10. **Reasons for Exclusion**	Report the reasons why participants were excluded after the run-in, including how many were excluded due to not meeting the adherence criterion used to include a participant in the main trial	*e.g. “A total of 118 participants were excluded following participation in the run-in phase. Reasons for exclusion were medication nonadherence (n* = *43), adverse events (n* = *31), failure to comply with protocol procedures (n* = *21), and withdrawal of consent (n* = *23).“*
11. **Baseline Characteristics of Excluded Participants**	Report whether the baseline characteristics of participants excluded after the run-in differed from those who proceeded to the main trial	If the participants that are excluded after the run-in are systematically different from those proceeding to the main trial, the external validity of the trial may be impacted. For instance, if there is a tendency for excluded participants to be older or higher BMI, the trial results may not generalize well to the broader population. Alternatively, if the baseline characteristics of participants that are excluded after the run-in are similar to those proceeding to the main trial, it should be explicitly stated *e.g. “The baseline characteristics of participants excluded after participation in the run-in were comparable with those proceeding to the main trial.”*

## Discussion

Our study produced the Measurement, Analysis and Reporting of Medication Adherence during the Run-in phase of clinical trials (MARMAR) guideline, which is the first guideline developed specifically for the run-in phase of clinical trials. The MARMAR guideline seeks to support best practice in the conduct and reporting of trial run-in phases, addressing a critical gap in existing guidance. MARMAR is supported by an accompanying user guide to assist researchers in the effective application of the guideline in practice.

Run-in periods are generally accepted by regulatory authorities when appropriately justified and when they do not compromise the integrity of the randomized comparison. This is exemplified by The FDA’s guidance for Enrichment Strategies for Clinical Trials describing run-in phases as a method to enhance adherence to trial interventions [3]. Nevertheless, a significant gap remains: the purpose, conduct, and impact of run-ins are often insufficiently documented, making it difficult to assess their potential for introducing selection bias or affecting trial interpretability and generalisability [16]. In part, this is a consequence of a lack of clear guidance from regulators, good practice guidelines or international standards in clinical trials.

Assessment of medication adherence in a run-in trial provides benefit in identifying participants unlikely to be adherent to study medications [4]. MARMAR has potential application for trials by providing guidance for measuring, analysing, and reporting adherence during trial run-in phases. Importantly, because the run-in phase occurs before randomization, it does not affect the randomised comparison and therefore falls outside the scope of the estimand framework, which addresses intercurrent events arising after randomisation [25].

A general reporting guideline for medication adherence in clinical trials (EMERGE) has already been developed [24]. The Timelines-Events-Objectives-Sources (TEOS) methodological framework was also created to support researchers in constructing operational definitions of medication adherence [26]. However, like the EMERGE guideline, the TEOS framework was not designed to specifically address the run-in phase of clinical trials and is therefore not directly applicable in this context. Furthermore, previous research has identified biases in adherence research, including those associated with different adherence measurement methods and metrics [27], and developed tools to identify biases impacting adherence research, namely the Risk of Bias tool for Interventional Adherence Studies (RoBIAS) and Risk of Bias tool for Observational Adherence Studies (RoBOAS). We therefore acknowledge that the MARMAR guideline should be applied in conjunction with the most unbiased methods and metric available, where possible.

Our study adopted methods comparable to those used in the development of EMERGE, with both drawing on the results of a literature review to inform a Delphi study. Although alternative approaches to reporting guideline development exist, including methods based on focus groups and consensus meetings, the Delphi method is increasingly regarded as the gold standard for developing reporting guidelines and we therefore chose this approach [17]. For consistency, we largely aligned our definition of consensus and criteria for expert selection with EMERGE, although we used a different Likert scale, and whilst EMERGE only recruited medication adherence experts, our study acknowledged the context of use of run-in phases by also including experts in trials methodology, regulatory drug trials, as well as public and patient involvement. Unlike EMERGE, which focuses solely on reporting, the MARMAR guideline also incorporates methodological considerations. This was a deliberate choice aimed at improving the conduct of run-in phases.

In developing the MARMAR guideline, we followed established best practice guidance for conducting Delphi studies, which included the RAND methodological guidance [22]. Consistent with this guidance, we clearly defined our consensus criteria, specified expert eligibility, and outlined the anticipated number of rounds as stopping criteria prior to survey administration, thereby reducing bias. Our explicit definition of “experts” further aligns with the RAND Guidance.

Strengths of this study include its novel focus on medication adherence during trial run-in phases, which makes an original contribution to the research area and to the conduct and reporting of run-ins generally, being the first and only guideline specific to this context. Development of the initial items was evidence-driven, informed by the results of a systematic review [16]. Additional strengths include the relatively high number of participants during the first Delphi round and the response rate for the second round. Moreover, the diversity of the expert panel, comprising international representatives from a range of disciplines and sectors of employment, strengthens the applicability of the final guideline.

Recruiting both experts in clinical trial methodology and medication adherence presented a unique challenge, as those experienced in methodological research or regulatory trials were not necessarily familiar with adherence research, and vice versa. Nonetheless, including all three expert groups was considered essential, as it broadened perspectives and strengthened the quality of feedback. Incorporating PPI contributors into the Delphi process also enhanced the relevance and overall quality of the research [28]. In the first Delphi round, consensus was reached for 28 of the 29 items. Re-rating the remaining item in the second round, as originally planned, would therefore have been redundant. Instead, the second round was adapted to ask participants to rate items as essential or desirable and to provide feedback on wording.

A further challenge arose from the overlap between methods and reporting items, which often shared similar wording and sentence structures. Presenting these items clearly within a single survey to ensure a clear distinction required careful consideration, but may nonetheless be misinterpreted by study participants. We could not guarantee that all participants interpreted each item in exactly the same way, although a principal objective of the Delphi technique is to refine items to improve comprehensibility. Additional limitations of the study include not knowing the characteristics of respondents in round 2 and therefore whether representativeness influenced any change in expert opinions, and not excluding the presence of undisclosed conflicts of interest. We were unable to use 9-point Likert scales as recommended by the Grading of Recommendations, Assessment, Development and Evaluations (GRADE) scale working group [29], because of incompatibility with our survey software. Although shorter scales may provide less granularity than 9-point formats, they are more widely used in Delphi studies as they require clearer agreement among participants to achieve consensus [30].

## Conclusions

As the first guideline developed specifically for the run-in phase of clinical trials, the MARMAR guideline provides an important foundation for improving both the methodological rigour and transparency of reporting of clinical trial run-in phases. MARMAR should serve to inform regulatory standards concerning trial run-in phases, and used alongside existing guidelines and frameworks to improve clinical trial methods, conduct and reporting.

## Supplementary Information

Additional file 1. Supplementary Tables 1 and 2.

## Data Availability

The protocol for the Delphi study is available on the Open Science Framework. The raw Delphi study data are available from the corresponding author on reasonable request.

## Abbreviations

MARMAR: Guideline for the Measurement, Analysis, and Reporting of Medication Adherence during the Run-in phase of clinical trials
ESPACOMP: The International Society for Medication Adherence
EQUATOR: Enhancing the QUAlity and Transparency Of health Research
FDA: United States Food and Drug Administration
EMA: European Medicines Agency
MHRA: Medicines and Healthcare Products Regulatory Agency
CONSORT: Consolidated Standards of Reporting Trials
GCP: Good Clinical Practice
EMERGE: ESPACOMP Medication Adherence Reporting Guideline
DELPHISTAR: Delphi studies in social and health sciences — Recommendations for an interdisciplinary standardized reporting
PPI: Patient and public involvement
TMRP: Trials Methodology Research Partnership
UK CRC CTUs: United Kingdom Clinical Trial Collaboration Clinical Trial Units
PSI: Statisticians in the Pharmaceutical Industry
TEOS: Timelines-Events-Objectives-Sources
RoBIAS: Risk of Bias tool for Interventional Adherence Studies
RoBOAS: Risk of Bias tool for Observational Adherence Studies
GRADE: Grading of Recommendations, Assessment, Development and Evaluations scale
