# Supplementary material for: Guideline for the Measurement, Analysis and Reporting of Medication Adherence during the Run-in phase of clinical trials (MARMAR)
Source: Trials. 2026 Apr 16;27:394. doi: 10.1186/s13063-026-09717-0 (PMC13202867; doi:10.1186/s13063-026-09717-0)
Supplement: Supplementary file 1 — Additional file 1. Supplementary Tables 1 and 2. [file 13063_2026_9717_MOESM1_ESM.docx]

Supplementary Table 1 – The MARMAR methods domain

| Theme | Item | Checklist |
| --- | --- | --- |
| 1. Adherence-Based Eligibility | If the run-in phase is used to identify participants who are adherent, clearly specify the adherence criteria that determine eligibility for inclusion in the main trial. |  |
| 1. Objective Progression Criteria | Decisions to progress participants from the run-in phase to the main trial based on medication adherence should be guided by a pre-specified metric, rather than left solely to investigator discretion. |  |
| 1. Appropriate Measurement Methods | The method(s) used to measure adherence during the run-in should be appropriate to the phase of adherence being studied, the dosing regimen, and the mode of administration. |  |
| 1. Risk of Bias and Imprecision | When selecting an adherence measurement method(s) for the run-in phase, assess the potential for bias and imprecision in relation to factors such as acceptability, practicability, feasibility, and cost. Consider risks such as low sensitivity/specificity, recall bias, and pill dumping. |  |
| 1. Impact of adherence monitoring on participant behaviour | Consider whether the chosen adherence measurement method(s) might itself influence participant behaviour (i.e. the Hawthorne effect)*,* and account for this in the trial design. |  |
| 1. Observation Period | Justify the duration of the observation period over which adherence is measured during the run-in. |  |
| 1. Adherence Summary Metrics | Justify the individual-level metric(s) used to summarise adherence during the run-in *(e.g. proportion of doses taken, number of days with correct dosing, questionnaire scores).* |  |
| 1. Data Management | Individual-level adherence data collected during the run-in phase should be treated consistently with other trial data, in accordance with the most recent ICH Good Clinical Practice guideline (E6 R3 or future iterations). |  |
| 1. Statistical Analysis | Justify the statistical methods used to analyse adherence data from the run-in phase |  |

Supplementary Table 2 – The MARMAR reporting domain

| Theme | Item | Reported on line/page number: |
| --- | --- | --- |
| 1. Purpose of Adherence Measurement | Clearly report the purpose of measuring adherence during the run-in phase. |  |
| 1. Eligibility Criteria for Adherence-Based Inclusion | If the run-in is used to select adherent participants for the main trial, report the specific adherence criterion used to define eligibility. |  |
| 1. Implications of Adherence-Based Selection | If adherence during the run-in is used to determine inclusion in the main trial, report the potential implications of this selection. |  |
| 1. Adherence Measurement Methods | Report the method(s) used to measure adherence during the run-in *(e.g. electronic monitoring, blood sampling, pill counts, self-reports).* |  |
| 1. Limitations and Biases of Measurement Methods | Report potential limitations of the adherence measurement method(s), including risks of bias and imprecision *(e.g. low sensitivity/specificity, recall bias, pill dumping, interviewer bias, non-response bias),* as well as whether the method may itself influence participants’ adherence behaviour. |  |
| 1. Observation Period | Report the duration of the observation period over which adherence was measured during the run-in. |  |
| 1. Adherence Summary Metrics | Report the metric(s) used to summarise adherence at the participant level during the run-in *(e.g. proportion of doses taken, days with correct dosing regimen, questionnaire scores),* and assess its limitations. |  |
| 1. Statistical Analysis | Report the results of the statistical analysis of adherence data collected during the run-in. |  |
| 1. Participant Flow | Report the number of participants enrolled into the run-in, excluded following the run-in, and those who proceeded to the main trial. Include this information in the study’s CONSORT diagram. |  |
| 1. Reasons for Exclusion | Report the reasons why participants were excluded after the run-in, including how many were excluded due to not meeting the adherence criterion used to include a participant in the main trial. |  |
| 1. Baseline Characteristics of Excluded Participants | Report whether the baseline characteristics of participants excluded after the run-in differed from those who proceeded to the main trial. |  |
